# Hepatitis E Virus in Pork Production Chain in Czech Republic, Italy, and Spain, 2010

**DOI:** 10.3201/eid1808.111783

**Published:** 2012-08

**Authors:** Ilaria Di Bartolo, Marta Diez-Valcarce, Petra Vasickova, Petr Kralik, Marta Hernandez, Giorgia Angeloni, Fabio Ostanello, Martijn Bouwknegt, David Rodríguez-Lázaro, Ivo Pavlik, Franco Maria Ruggeri

**Affiliations:** Istituto Superiore di Sanità, Rome, Italy (I. Di Bartolo, G. Angeloni, F.M. Ruggeri);; Instituto Tecnológico Agrario de Castilla y León, Junta de Castilla y León Valladolid, Spain (M. Diez-Valcarce, M. Hernandez, D. Rodríguez-Lázaro);; Veterinary Research Institute, Brno, Czech Republic (P. Vasickova, P. Kralik, I. Pavlik);; University of Bologna, Bologna, Italy (F. Ostanello);; and National Institute for Public Health and the Environment (RIVM), Bilthoven, the Netherlands (M. Bouwknegt)

**Keywords:** Hepatitis E virus, swine, pork food chain, molecular detection, reverse transcriptase polymerase chain reaction, zoonoses, food safety, viruses, Czech Republic, Italy, Spain

## Abstract

We evaluated the prevalence of hepatitis E virus (HEV) in the pork production chain in Czech Republic, Italy, and Spain during 2010. A total of 337 fecal, liver, and meat samples from animals at slaughterhouses were tested for HEV by real-time quantitative PCR. Overall, HEV was higher in Italy (53%) and Spain (39%) than in Czech Republic (7.5%). HEV was detected most frequently in feces in Italy (41%) and Spain (39%) and in liver (5%) and meat (2.5%) in Czech Republic. Of 313 sausages sampled at processing and point of sale, HEV was detected only in Spain (6%). HEV sequencing confirmed only g3 HEV strains. Indicator virus (porcine adenovirus) was ubiquitous in fecal samples and absent in liver samples and was detected in 1 slaughterhouse meat sample. At point of sale, we found porcine adenovirus in sausages (1%–2%). The possible dissemination of HEV and other fecal viruses through pork production demands containment measures.

## Abstract 2

Processing does not substantially abate endogenous virus.

Human hepatitis E is endemic worldwide, particularly in Asia, where large waterborne outbreaks have been reported (*1*). Seroprevalence of hepatitis E virus (HEV) is >60% in rural southern People’s Republic of China (*2*) and 4%–10% in western Europe (*3*) and the United States (*4*). In these areas, hepatitis E occurs mostly as sporadic cases (*5**–**7*), but epidemics also have been described (*8*). Most cases in Europe have been linked to genotype 1 (g1) virus and associated with travel to g1-endemic areas. However, autochthonous human infections are increasing in Europe and in other industrialized countries (*5**,**6**,**9*). Of the 4 genotypes affecting humans, genotype 3 (g3) is the main HEV genotype also circulating among pigs in Europe (*10*) and human infections are observed sporadically worldwide (*11**,**12*).

Several reports indicate that HEV can be transmitted through zoonotic and foodborne pathways, including through consumption of raw and undercooked liver, meat, or sausages from domestic pigs, wild boar, and deer (*8**,**13**,**14*). Several investigations have shown that farmed domestic pigs are widely infected with and shed g3 HEV in Europe. Studies conducted in Spain (*8**,**13**,**14*), Italy (*15*), and France (*16*) have detected HEV genomic RNA in livers of pigs of slaughtering age, indicating that HEV-contaminated food might reach supermarkets (*17*). In butcher shops in the Netherlands (*18*) and Germany (*19*), ≈6.5% and ≈4%, respectively, of pork livers contained HEV, which raises concern about the potential for direct transmission through contact with or consumption of contaminated food.

Despite the large widespread distribution of HEV-shedding pigs and the possible role of farmed pigs as the main virus reservoir, the number of human hepatitis E cases in Europe remains low, suggesting inefficient virus transmission or lower pathogenicity of swine g3 strains than of g1 strains for humans. Because g3 HEV is common in pigs but rare in humans, humans are postulated to not be a main host for g3 virus replication (*11**,**20*). Nonetheless, a possibly large underestimation of HEV spread in humans cannot be excluded because of asymptomatic cases, inadequate diagnostics, and scarce medical attention (*21*). Mansuy et al. suggested inadequacy of previous diagnostic methods and recently found unprecedentedly high HEV seroprevalence among blood donors in France (*22*).

Relatively few studies of foodborne human hepatitis E are available (*8**,**21**,**23*), making evaluation of HEV-associated risks difficult. Investigation of HEV throughout the pork production chain from farm to point of sale is needed to highlight areas of risk and proper control. A recent report from European Food Safety Authority biohazard experts (*24*) underscored an urgent need for integrated studies on HEV circulation, performing farm-to-table integrated risk assessment. For other foodborne pathogens, such studies comprise quantitative microbial risk assessment on the basis of exposure and dose-response models (*25*). Unfortunately, for HEV, quantitative approaches are hardly accessible because of the absence of reliable cell culture systems for viral infectivity titration.

We aimed to assess HEV prevalence in the pork production chain from slaughterhouse to point of sale in Czech Republic, Italy, and Spain during 2010 in the framework of the FP7 VITAL (Integrated Monitoring and Control of Foodborne Viruses in European Food Supply Chains) project (www.eurovital.org). This systematic multicountry investigation of domestic swine HEV was conducted by using standardized molecular approaches, including reverse transcription quantitative PCR (RT-qPCR) detection, process, and internal amplification controls (IACs) and proper fecal viral indicators (porcine adenovirus [PAdV]).

## Materials and Methods

### Sampling Strategy

Samples were taken at perceived critical points for virus contamination. They were identified from Hazard Analysis and Critical Control Point System audit principles-based questionnaires (K. Willems and R. Moloney, pers. comm.) completed in each premise and analyzed by VITAL food-safety management and risk assessment experts (M. Bouwknegt and A. De Roda Husman, pers. comm.).

### Samples

A total of 113 fecal, 112 liver, and 112 meat (lingual muscle) samples from 113 healthy pigs (*Sus scrofa* subsp. *domestica*) were collected in slaughterhouses from Czech Republic, Italy, and Spain during 2010 (Table 1). Samples originated from 4 pig farms per country. Packaged sausages were sampled in processing sites and supermarkets in Italy and Spain (128 and 93 samples, respectively) and in 8 supermarkets in Czech Republic (92 samples).

**Table 1:** Detection of HEV and indicator virus PAdV in samples from the pork production chain, Czech Republic, Italy, and Spain, 2010*

Production stage and sample source	Virus	Czech Republic			Italy			Spain			All	
		No. tested	No. (%) positive		No. tested	No. (%) positive		No. tested	No. (%) positive		No. tested	No. (%) positive
Slaughterhouse												
Feces	HEV	40	1 (3)†		34	14 (41)		39	15 (38)†		113	30 (27)
	PAdV	40	39 (98)		34	31 (91)		39	35 (90)		113	105 (93)
Liver	HEV	40	2 (5)†		33	2 (6)		39	1 (3)†		112	5 (4)
	PAdV	40	0		33	0		39	0		112	0
Meat	HEV	40	1 (3)		33	2 (6)		39	0		112	3 (3)
	PAdV	40	0		33	1 (3)‡		39	0		112	1(1)
Processing/points of sale: sausage	HEV	92	0		128	0		93	6 (6)		313	6 (2)
	PAdV	92	1 (1)		128	1 (1)		93	2 (2)‡		313	4 (1)

*HEV, hepatitis E virus; PAdV, porcine adenovirus. †Samples originated from the same animal. ‡Sample negative for HEV.

Additional ad hoc samples were collected during fact-finding visits to production farms, processing plants, and points of sale (Table 2). Briefly, 73 samples were collected from working surfaces and cutting tools (swabs from knife, belt surface, and meat mincer) from slaughtering areas (10 samples), processing areas (19 samples), and points of sale (12 samples) and from workers’ hands (20 samples) and workers’ toilets (12 samples). In Czech Republic, 6 effluent water samples from slaughterhouses also were examined.

**Table 2:** Detection of HEV and indicator virus PAdV in swabs in the pork production chain, Czech Republic, Italy, and Spain, 2010*

Production stage (area), sample type	No. tested	Positive, no. (%)	
		HEV	PAdV
Production (slaughterhouse: carcass dissection and liver removal)			
Water effluents	6	0	0
Workers’ hands and aprons	7	4 (57)	5 (71)
Working surfaces	10	6 (60)	6 (60)
Processing (skin removal and sausage preparation)			
Workers’ hands	7	2 (29)	1 (14)
Working surfaces	19	4 (21)	0
Points of sale			
Workers’ hands and gloves	6	1 (17)	0
Working surfaces	12	1 (8)	0
Hand wash basin tap and toilet edge	12	1 (8)	1 (8)
All samples	79	19 (24)	13 (16)

*HEV, hepatitis E virus; PAdV, porcine adenovirus.

### Sample Process Control Virus

Murine norovirus 1 (MNV-1) was used as sample process control virus (SPCV). A single batch with MNV-1 at the concentration of 4.7 × 10^7^ PFU/mL was prepared and used by all collaborating institutes throughout the study (*26*).

### Virus Concentration and Nucleic Acid Isolation

#### Pig Feces

Feces (>1 g) were collected aseptically. A total of 250 mg of sample in 15-mL centrifuge tubes were suspended in 2.25 mL phosphate-buffered saline containing gentamycin (10 mg/mL), and 10 μL SPCV (4.7 × 10^5^) was added. Suspensions were vortexed for 60 s and centrifuged at 3,000 × *g* for 15 min. Supernatants were immediately used for nucleic acid isolation or stored at –70°C. Nucleic acid was extracted by using a QIAamp Viral RNA Mini Kit (QIAGEN, Hilden, Germany) according to the manufacturer’s instructions. Final elution was performed 2× with 50 µL elution buffer, resulting in a 100-µL nucleic acid extract, for immediate testing or −70°C storage.

#### Pork Liver, Meat, and Sausages

Liver and meat or sausage samples were collected (1 cm^3^ from 3 different locations) and stored in sterile plastic bags. According to the method of Bouwknegt et al. (*18*), samples were finely chopped and homogenized in an RNase-free mortar with 4 mL of Buffer RLT (RNeasy Midi Kit, QIAGEN) containing 1:100 β-mercaptoethanol. A total of 250 mg homogenate was transferred into microcentrifuge tubes containing 1 mL RLT buffer, 2.5 g sterile 1-mm zirconia beads (BioSpec Products, Inc., Bartlesville, OK, USA), and 10 μL SPCV (4.7 × 10^5^). Tubes were applied to a mechanical disruptor (Ribolyser-Cell-Disrupter, Hybaid Ltd., Ashford, UK) for two 40-s/4-m s^–1^ cycles. After centrifugation (10,000 × *g*, 20 min, 2×), 800 µL of resulting supernatants were immediately processed by RNeasy Midi Kit or freeze-stored. Nucleic acid extracts (300 µL) were assayed immediately or freeze-stored.

#### Workers’ Hands and Surfaces

Workers’ hands and surfaces were sampled by using sterile moistened swabs, and samples were stored in 5 mL of 10 mg mL^–1^ gentamicin-containing phosphate-buffered saline in plastic tubes. Unwashed hands were sampled immediately before lunch or afternoon coffee break. For surfaces, 10-cm^2^ areas were rubbed. Liquids were decanted from swab containers into 50-mL centrifuge tubes containing 10 μL SPCV (4.7 × 10^5^). Suspensions were vortexed and centrifuged (3,000 × *g*, 5 min), and supernatants were used immediately or freeze-stored. Nucleic acids were extracted by NucliSENS miniMAG Kit (bioMérieux, Marcy l’Etoile, France) and eluted 2× with 50 µL elution buffer.

### RT-qPCR

Nucleic acids were assayed undiluted and diluted 10-fold by performing RT-qPCRs in duplicate. All reaction mixes included an IAC (*27*). All RT-qPCRs were in duplex format, targeting specific viruses (MNV-1, HEV, PAdV) and IACs labeled with FAM (6-carboxy fluorescein) and VIC (Applied Biosystems, Foster City, CA, USA) probes, respectively. All tests included virus- and IAC-negative controls.

#### PAdV RT-qPCR

A duplex RT-qPCR was used as described (*28*), including IACs and a carryover contamination prevention system using uracil-N-glycosylase (Roche Molecular Diagnostics, Manheim, Germany). Reactions contained 1× TaqMan Universal PCR Master-Mix (Life Technologies, Branchburg, NJ, USA), 0.9 µM primers, 0.225 µM PAdV TaqMan probe (FAM-labeled), 50 nM IAC probe (VIC-labeled), and 100 copies of PAdV IAC. Ten microliters of nucleic acid extract were added to 25-µL final reaction volumes. Thermocycling conditions were 2 min at 50°C and 10 min at 95°C, followed by 45 cycles of 15 s at 95°C and 1 min at 60°C.

#### HEV RT-qPCR

A 1-step duplex RT-qPCR was used (*29*) and included IACs. Reactions contained 1× RNA UltraSense reaction mix (Life Technologies), 0.25 µM primers, 0.1 µM probe HEV-P (FAM-labeled), 50 nM IAC probe (VIC-labeled), 1× ROX reference dye, 1 µL RNA UltraSense enzyme mix, and 300 HEV IAC copies. Ten microliters of nucleic acid extracts were added to 20 µL final volumes. Thermocycling conditions were 15 min at 50°C and 2 min at 95°C, followed by 45 cycles of 10 s at 95°C, 20 s at 55°C, and 15 s at 72°C.

#### MNV-1 RT-qPCR

A 1-step duplex RT-qPCR was adopted (*30*), including IACs. Reaction contained 1× RNA UltraSense reaction mix, 0.2 µM primers, 0.2 µM probe minor groove binder–open reading frame (ORF) 1/ORF2 (FAM-labeled), 50 nM IAC probe (VIC-labeled), 1× ROX reference dye, 1 µL RNA UltraSense enzyme mix, and 600 MNV-1 IAC copies. Ten microliters of nucleic acid extract were added (final reaction volume 20 µL). Thermocycling conditions were 15 min at 50°C and 2 min at 95°C, followed by 45 cycles of 15 s at 95°C and 1 min at 60°C.

### Data Reporting and Interpretation

For proper results interpretation, we considered 4 signals: 1) target virus, 2) SPCV, 3) target IAC, and 4) SPCV IAC (*31*). With cycle threshold (C_t_) <45, independently of corresponding IAC C_t_, the PCR result was considered positive. With C_t_>45 and corresponding IAC C_t_<45, results were interpreted as negative. When both targets and corresponding IACs showed C_t_>45, reactions were considered failed. When >1 replicate target assay (HEV or PAdV) was positive, the sample was considered positive. Absence of SPCV and its IAC signals indicated preamplification processes (virus concentration and extraction) failure (*31*). In the presence of SPCV, SPCV IAC, and target IAC signals, target virus signal absence was conclusively indicating test negative result.

### HEV Genotyping

Positive HEV samples were sequence-analyzed amplifying 2 ORF2 regions (348- and 121-bp fragments) (*32**,**33*). Sixteen sequences obtained were examined in GenBank (www.ncbi.nlm.nih.gov/genbank). The 5 shorter 100-bp fragments (3 fecal samples in Italy and 1 liver and meat sample in Czech Republic) were used only to identify genotype or confirm longer sequences. The 11 longer sequences (300 bp) from 4 fecal samples in Italy (GenBank accession nos. JN861803, JN861804, JN861805, JN861806) and 7 sequences from 5 sausages and 2 environmental swabs in Spain (GenBank accession nos. JN903913, JN903914, JN903915, JN903916, JN903917, JN903918, JN903919) also were used for HEV genotyping and subgenotyping. We performed phylogenetic analyses with Bionumerics v6 (Applied Maths, Kortrijk, Belgium) by using the neighbor-joining method with 1,000 replicates with Kimura-2 correction factor.

## Results

### HEV in Pork Products

We detected HEV RNA in all pork production chain sites in investigated countries, with some differences (Table 1). Overall, HEV RNA was detected in >1 samples (feces, liver, meat) from 36 (32%) of 113 pigs examined at slaughterhouses for which all sample types were collected (Table 1). HEV RNA was detected frequently in slaughterhouse samples in Italy and Spain, i.e., 18 (53%) positive samples from 34 animals and 15 (38%) of 39, respectively (Table 1), whereas in Czech Republic, HEV RNA prevalence at slaughterhouses was remarkably lower, i.e., 3 (8%) positive samples from 40 animals. Pig feces showed highest HEV RNA presence (27%), followed by liver (4%) and meat (3%) (Table 1).

Sausage samples from Italy and Spain were collected from processing plants of the same company slaughtering animals or from same company products in local supermarkets. Sausages sampled in Czech Republic were obtained from randomly chosen supermarkets. HEV was detected in 6 (6%) of 93 samples in Spain, whereas 0 of 220 sausages in Czech Republic or Italy were positive.

### PAdV in Pork Products

To evaluate possible fecal contamination, PAdV DNA presence (*34*) was determined for all samples assayed for HEV. PAdV was highly prevalent in feces (90%–98%) in investigated countries (Table 1). None of 112 liver samples were PAdV positive, and only 1 of 112 meat samples was PAdV positive, in Italy. In addition, 4 (1%) of 313 sausages (2 from Spain, 1 each from Czech Republic and Italy) were positive for PAdV (Table 1).

### Environmental Samples

We collected 41 surface swabs from working surfaces, meat mincers, knives, and other working items at the 3 pork production chain sites. Overall, swab samples were positive for either HEV (11 [27%] of 41) or PAdV (6 [15%] of 41) (Table 2). HEV-positive samples were found more frequently at slaughterhouse (6 of 10) than at processing and points of sale (4 [21%] of 19 and 1 [8%] of 12, respectively) sites, and PAdV was found only in slaughterhouse samples (6 of 10). At slaughterhouses, positive swabs (3 knives, 2 floor, 1 belt surface) contained both HEV and PAdV, indicating potential fecal contamination during slaughtering steps, whereas 0 of 5 HEV-positive samples at processing and points of sale sites was positive for PAdV, disproving possible fecal cross-contamination during later production phases (Table 2). A total of 20 swab samples were taken from workers’ hands, gloves, or aprons along the production chain. Overall results were similar to those for working surfaces in slaughtering premises; in fact, 5 (71%) of 7 samples were positive for both HEV and PAdV. Moreover, PAdV was detected in 1 of 2 HEV-positive samples at processing sites (Table 2). Finally, HEV or PAdV was detected in 1 (8%) of 12 toilet swab samples collected at points of sale. The 6 Czech Republic slaughterhouse effluent samples were negative for both PAdV and HEV.

### Sequence Analysis

**Figure A1:**
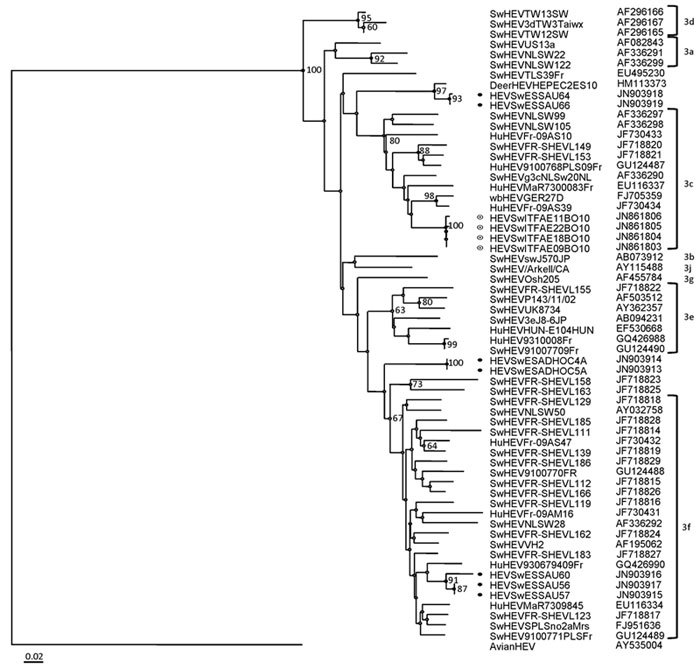
Phylogenetic tree constructed with the Bionumerics version 6 software (Applied Maths, Kortrijk, Belgium) by the neighbor-joining method on the basis of partial nucleotide sequences of open reading frame 2, with avian hepatitis E virus as an outgroup (GenBank accession no. AY043166). Samples are from Italy and Spain, 2010. Bootstrap values of >60% are indicated. ● indicates sequences from Spain; ◉ indicates sequences from Italy. Subtypes of genotype 3 HEV strains are indicated. Scale bar indicates number of substitutions per site.

HEV-positive samples were genotyped and sequenced to determine possible animal or human origin of the virus. A total of 9 samples (4 from Italy, 5 from Spain) yielded ≈300-bp sequences and were compared with HEV sequences in public databases. All HEVs belonged to g3. The 4 HEV-positive samples (HEVSwITFAE09BO10, HEVSwITFAE18BO10, HEVSwITFAE22BO10, HEVSwITFAE11BO10) in feces from slaughterhouses in Italy originated from the same herd and belonged to subtype g3c, sharing 99.4%–100% identical nucleotides. Three of 5 sequences from sausage in Spain belonged to subtype g3f (HEVSwESSAU56, HEVSwESSAU57, HEVSwESSAU60), whereas 2 additional g3 strains (HEVSwESSAU64, HEVSwESSAU66; 99.5% identity) could not be assigned to specific subtypes (Figure A1), although their sequences were closer to subtype g3c. Two sequences from swabs collected in Spain (HEVSwESADHOC4A, HEVSwESADHOC5A) also belonged to g3f, showing 100% reciprocal nucleotide identity and 89% identity with g3f strains from sausages. Sequences for g3 subtypes from Italy and Spain exhibited <85% nucleotide identity, suggesting circulation of different strains in these countries.

Shorter sequences (121 bp, ORF2) also were obtained (*33*) from 3 fecal samples in Italy (100% identity), and 2 additional identical sequences were obtained from liver and meat at a slaughterhouse in Czech Republic. All were confirmed as g3 swine HEV, but further subtyping was not possible because of short sequence length.

## Discussion

Pork is a major food source worldwide (*10*), and HEV is widespread among farmed swine and can be transmitted zoonotically, including through pork products (*5**,**10*). We investigated HEV presence throughout the pork production chain in 3 European countries from pigs entering slaughterhouses through processing to retail stores.

To optimize detection sensitivity, in our sampling strategy we assumed low HEV prevalence in pork products and environmental surfaces (*17**,**35*) and involved 3 laboratories. In addition to liver, we selected sausage because it is handled by consumers and is a blend of different meat and slaughtered animals. To maintain consistent results among countries and sample treatment, we validated standardized sampling and molecular procedures by ring test (*36*), including IAC and sample process controls (*26**,**27**,**31*).

Samples analyzed throughout the pork production chain in Italy and Spain were from the same herds from farm to retail sale. In Czech Republic, more points of sale were sampled, thus representing a larger animal population. HEV prevalence in pig feces was similar in Italy and Spain (41% and 38%, respectively), reflecting previous data in these and other European countries (*10*). Conversely, only 3% of pigs from Czech Republic shed HEV. Because of shared protocols and controls, this difference cannot be attributed to different diagnostic sensitivity among partners, which otherwise detected HEV in similar numbers of liver and meat samples.

Lower HEV shedding by pigs in Czech Republic might reflect different farming methods, such as animal housing and separation, herd size, slaughtering age, and/or environmental factors that possibly influence infectious HEV persistence, spread, and transmission. Previous data from Czech Republic (*37*) showed up to 40.0% HEV-positive bile samples from piglets, suggesting infection rates close to shedding rates reported for Italy and Spain. However, that study did not examine HEV fecal shedding, and pigs were only 2–3 months of age. Furthermore, varying prevalence of HEV in pig feces also has been reported in Italy and Spain (*15**,**38*), possibly reflecting differences in farm selection.

The absence of fecal HEV in pigs with HEV-positive liver or bile in Czech Republic suggests that bile concentration in the fecal mass was lower when samples were taken, as might be expected if pigs were fed long before reaching the slaughterhouse. This finding might also help explain the different fecal HEV positivity among countries.

We confirm broad HEV circulation within pig farms and HEV RNA in livers and other pork products (*8**,**17**,**18*). We found HEV prevalence in 3%–6% of liver samples at slaughter, similar to findings in the Netherlands (*18*) but somewhat less than in the United States (11%) (*17*). HEV RNA was present in meat samples only in Czech Republic and Italy (3% and 6%, respectively), whereas sausages were HEV positive only in Spain (6%). This finding might result from low sample numbers but also could reflect different methods for final product preparation by using different meat blends, fat, or liver intentionally or after unintentional cross-contamination.

HEV positivity markedly decreased from feces (27%) to liver (4%), meat (3%), and sausage (2%) but never disappeared during production. However, detection of HEV by RT-qPCR did not conclusively demonstrate viable virus and thus risks to consumers.

PAdV has been confirmed as a suitable indicator of swine fecal contamination during pork production (*28*). Although most pig feces in our study were PAdV positive (90%–98%), PAdV was never detected in liver and detected only occasionally in pork meat (1/33 samples in Italy) or sausage (4/313 samples, all 3 countries). Comparing HEV and PAdV findings, risks for cross-contamination of pork products with swine feces during preparation appear to be low but not absent.

Three of 112 pork meat samples tested were positive for HEV and 1 for only PAdV (Table 1). We have no proof of HEV replication in muscle, and finding HEV RNA in pork products probably reflects endogenous HEV particles in infected liver and/or viremic blood (*39*). Although liver and bile are usually removed before processing, the HEV genome sporadically detected in meat most likely represents cross-contamination of carcasses during slaughtering, which suggests a need for worker training.

PAdV detection in 1 meat sample and 4 sausages also indicates some fecal contamination during slaughtering, which was, however, similarly low in all countries. PAdV and HEV were not present in the same sausage samples from Spain, and PAdV was detected in fewer samples than was HEV (2 vs. 6), which argues against potential higher risks for fecal contamination in the food chain in Spain. The higher HEV prevalence in sausage in Spain than in Italy or Czech Republic is unclear and deserves further investigation.

The samples from food handlers and the environment in Italy and Spain also identified areas where procedures and information could be implemented. Detection of HEV and PAdV in 60% of floor and working surfaces and 57%–71% of hands and aprons of workers dissecting pigs indicates that the initial production areas (bleeding to evisceration) are at higher risk for fecal contamination and highlight possible hazards to workers.

In the cutting/slicing/chopping areas, we did not detect PAdV in fecal samples. However, HEV detection on hands and surfaces indicates that endogenous HEV can be spread during cutting of liver and meat in industrial premises, requiring cross-contamination control measures. Limited handling might instead explain the single detection of HEV on a butcher’s bench at point of sale.

The HEV detected from a supermarket personnel toilet was not genotyped. Thus, its possible origin, i.e., pig versus human, cannot be confirmed.

Our analysis of short sequences confirms presence of only g3 HEV. Sixteen sequences from Italy and Spain were subtyped; g3c was identified as the prevalent strain in Italy, and the less common g3f was noted only in Spain. Two identical HEV sequences in sausage from Spain might represent a novel g3 subtype, similar to a deer g3 HEV strain found in Spain in 2010 (*40*).

In conclusion, our study indicates that HEV is present throughout the pork production chain and that processing does not substantially abate endogenous virus. Consequently, consumers might purchase pork products that contain detectable HEV genome in up to 6.0% of instances, independent of source and country of origin, probably unrelated to fecal contamination during pork processing.

We cannot exclude the possibility that in some pork products HEV was infectious. However, HEV infectious dose for humans is unknown, and viral load in pork might not be sufficient to infect humans efficiently. Storage, processing, and blending of meat from HEV-positive and -negative animals (e.g., sausage) might substantially decrease risks for foodborne infection, possibly explaining why HEV food transmission in Europe seems relatively inefficient. However, consumers should eat only pork that has been thoroughly cooked, particularly liver, and avoid cross-contamination of surfaces and other food by handling pork products, especially offal.

This study addressed only fresh meat or sausage sold within few days of preparation. Future studies should be extended to other pork products, such as salami, which are eaten after short periods of curing and might still contain residual infectious virus.
